# High-throughput design of optoelectronic–ferroelectric heterostructure from materials to sensor–memory–computing devices

**DOI:** 10.1093/nsr/nwaf530

**Published:** 2025-11-26

**Authors:** Gaokuo Zhong, Jiaqi Yan, Mingkai Tang, Haoyue Deng, Yangchun Tan, Xiangli Zhong, Changjian Li, Zhen Fan, Jinbin Wang, Jiangyu Li

**Affiliations:** Changsha Semiconductor Technology and Application Innovation Research Institute, College of Semiconductors (College of Integrated Circuits), Hunan University, Changsha 430100, China; Shenzhen Institute of Advanced Technology, Chinese Academy of Sciences, Shenzhen 518000, China; Shenzhen Institute of Advanced Technology, Chinese Academy of Sciences, Shenzhen 518000, China; National-Provincial Laboratory of Special Function Thin Film Materials, School of Materials Science and Engineering, Xiangtan University, Xiangtan 411100, China; Shenzhen Institute of Advanced Technology, Chinese Academy of Sciences, Shenzhen 518000, China; National-Provincial Laboratory of Special Function Thin Film Materials, School of Materials Science and Engineering, Xiangtan University, Xiangtan 411100, China; Institute for Advanced Materials, South China Academy of Advanced Optoelectronics, South China Normal University, Guangzhou 510000, China; Shenzhen Institute of Advanced Technology, Chinese Academy of Sciences, Shenzhen 518000, China; National-Provincial Laboratory of Special Function Thin Film Materials, School of Materials Science and Engineering, Xiangtan University, Xiangtan 411100, China; National-Provincial Laboratory of Special Function Thin Film Materials, School of Materials Science and Engineering, Xiangtan University, Xiangtan 411100, China; Department of Materials Science and Engineering, Southern University of Science and Technology, Shenzhen 518000, China; Institute for Advanced Materials, South China Academy of Advanced Optoelectronics, South China Normal University, Guangzhou 510000, China; National-Provincial Laboratory of Special Function Thin Film Materials, School of Materials Science and Engineering, Xiangtan University, Xiangtan 411100, China; Department of Materials Science and Engineering, Southern University of Science and Technology, Shenzhen 518000, China

**Keywords:** ferroelectric, ferroelectric field-effect transistors, high-throughput, artificial synapses, sensing–memory–computing integration

## Abstract

Ferroelectric-based artificial synapses have emerged as fascinating candidates for the development of intelligent sensor–memory–computing (SMC) systems, thanks to the remarkable nonvolatile properties and abundant polarization states that ferroelectrics offer. However, simultaneously modulating the ferroelectric synapse through optical and electrical excitation is challenging. Herein, we propose a high-throughput strategy for designing optoelectronic co-modulated ferroelectric synapses. This strategy involves designing a ferroelectric field-effect transistor (FeFET) based on the Pb(Zr_0.2_Ti_0.8_)O_3_/InGaZnO (IGZO) heterostructure, which includes an IGZO homostructure, followed by high-throughput screening of IGZO materials that enable both optical and electrical modulation. The transistors with optoelectronic co-modulated synaptic functionalities are subsequently screened from a set of high-throughput FeFETs. Based on these optoelectronic co-modulated ferroelectric synapses, an artificial SMC system that can simultaneously sense and recognize images is constructed, achieving a high recognition accuracy of 88.42% and this SMC system simultaneously exhibits the advantages of reduced hardware overheads, fast speed and low power consumption. Our work introduces a novel strategy for designing multifunctional artificial synapses from materials to devices, which may represent a new paradigm in the development of high-performance SMC systems.

## Teaser

A high-throughput strategy for designing materials-to-device optoelectronic co-modulated ferroelectric synapses, highlighting the efficiency and exploratory potential of systematic high-throughput approaches.

## INTRODUCTION

**Figure 1.**
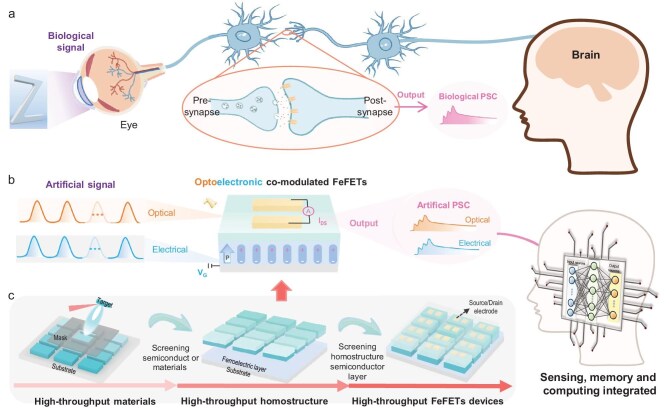
Optoelectronic co-modulated synapse. (a) Schematic of the biological sensor–memory–computing (SMC) system. (b) Schematic of the artificial SMC system with optoelectronic co-modulated FeFETs. (c) Flow chart of the high-throughput design of optoelectronic co-modulated FeFETs.

Neuromorphic computing systems, particularly sensor–memory–computing (SMC) systems, operate by using a massively parallel processing strategy and exhibit energy-efficient capabilities comparable to those of human brains [1–3]. This characteristic renders them highly promising for intelligent applications in a variety of fields, such as target tracking, image analysis and autonomous driving [4–6]. The ability of artificial synapses to mimic biological synapses and perform synaptic functionalities is very desirable in neuromorphic computing systems (Fig. 1a and b). Several types of materials have been explored for designing artificial synapses, including valence change, phase change, electrochemical metallization and ferroelectric [7–10]. Among these, ferroelectric materials have emerged as promising candidates for artificial synapses due to their remarkable nonvolatile properties and the abundant polarization states they offer [10–14]. In particular, ferroelectric field-effect transistors (FeFETs) utilize the gate terminal to modulate the conductance, enabling precise control over the synaptic weight [14–16]. Additionally, the extra terminal provides greater flexibility in programming and tuning the synaptic properties, enabling the implementation of diverse synaptic functionalities [16–18]. With the development of FeFET-based synapses, electrically modulated synaptic functions and artificial neural networks (ANNs, as shown in Fig. S1a) have been demonstrated in various FeFETs systems, such as BaTiO_3_/MoS_2_, Pb(Zr, Ti)O_3_/ZnO, HfLaO/ITO and SiO_2_/HfZrO*_x_*/InZnO*_x_* [10,19–21]. However, to process visual information, such FeFET synapses require additional photoelectric sensors to convert light signals into electrical signals. Alternatively, neuromorphic vision sensors with optically modulated synapses enable the preprocessing of visual information (Fig. S1b), but postprocessing units are needed to further process the data [22].

From an in-sensor computing perspective, if FeFET-based synapses are both electrically and optically modulatable (Fig. 1b), then they can be used to construct ANNs with an integrated sensor–memory–computing (SMC) architecture. Such SMC systems can realize the real-time perception and processing of visual information directly in the sensor (Fig. S1c), which can greatly reduce hardware overheads, time latency and energy consumption [5,23]. Nevertheless, the development of FeFET synapses capable of optoelectronic co-modulation remains a challenge due to the conflicting requirements of the physical properties within the FeFETs. As shown in Fig. S1d, the synaptic plasticity modulation processes, such as typical long-term potentiation (LTP), can be performed by redistributing the carriers in the semiconductor layer via progressively switching the ferroelectric polarization. This requires the semiconductor layer to have (i) a low oxygen-vacancy concentration to ensure a high-quality ferroelectric/semiconductor interface and (ii) a low carrier density to achieve significant carrier redistribution [24]. However, such a semiconductor layer is often insensitive to light [24,25], making it difficult to achieve the optical modulation of synaptic plasticity (as discussed in Fig. S1e). On the other hand, as shown in Fig. S1f, the use of a semiconductor layer with a high carrier density and a high concentration of oxygen vacancies enables the optical modulation of synaptic plasticity because of the persistent photoconductivity associated with the oxygen vacancies [26]. However, using such a semiconductor layer hinders the electrical modulation [27] (as discussed in Fig. S1g). To address the conflicting requirements, it is highly desirable to develop a systematic strategy that enables the rapid screening of material parameters and heterostructure combinations. A pulsed-laser-deposition-based high-throughput (HT-PLD) technique has recently emerged as an efficient route for accelerating thin-film optimization. For example, compositional and thickness screening of ferroelectric films enables the correlation between material properties, device performance and synaptic functionality [28–30]. Here, we propose a high-throughput strategy that establishes a quantitative material–device–system correlation for developing optoelectronic co-modulated FeFET synapses, in which the FeFET is constructed based on a Pb(Zr_0.2_Ti_0.8_)O_3_ (PZT)/InGaZnO (IGZO) heterostructure due to its outstanding ferroelectricity and tunable transport properties. As illustrated in Fig. S2, this strategy includes the high-throughput screening of optically tunable IGZO thin films, optimization of the ferroelectric PZT thickness to ensure robust polarization at a low switching voltage (Fig. S3) and the subsequent high-throughput evaluation of the IGZO/PZT heterostructures at varied thickness ratios. Based on this approach, FeFET synapses with reliable optoelectronic co-modulated plasticity are successfully realized. Unlike non-ferroelectric IGZO devices, which rely on volatile defect-mediated processes, our FeFETs achieve stable, nonvolatile conductance modulation via ferroelectric polarization switching [31,32]. Based on this high-throughput strategy, the FeFET synapses with optoelectronic co-modulated synaptic plasticity are successfully created (Fig. 1c). These FeFETs are further used to construct an SMC system, which achieves high recognition accuracy (88.42%). More importantly, the SMC system can simultaneously sense and recognize images, whereas the MC counterpart still needs additional photoelectric sensors, thus rendering the SMC system simpler yet faster and more energy-efficient than the memory-computing (MC) counterpart. This multistep high-throughput strategy is generally applicable to a wide range of heterostructure systems, including both lead-based and lead-free ferroelectrics, semiconductors, dielectrics and layered oxides. This work provides a novel strategy for designing artificial synapses with multifunctionalities, which may facilitate the development of intelligent SMC systems.

## RESULTS AND DISCUSSION

**Figure 2.**
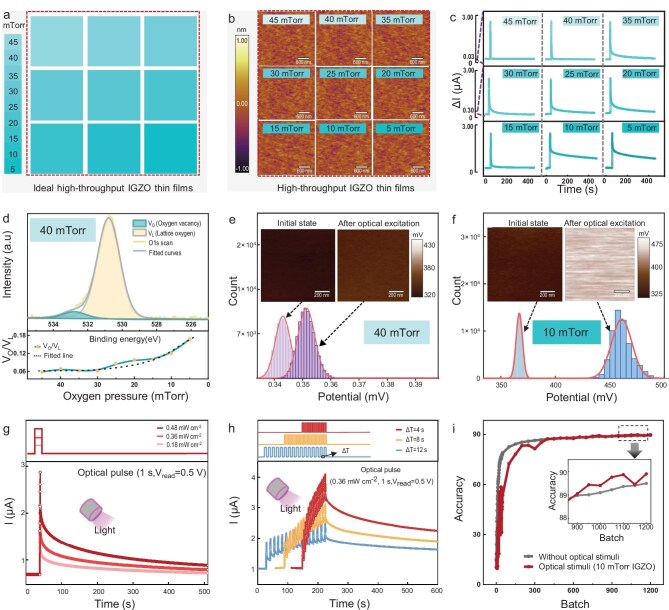
High-throughput screening of IGZO thin films. (a) Schematics of the high-throughput IGZO thin films with growth oxygen pressures ranging from 45 to 5 mTorr. (b) Topography of these high-throughput IGZO thin films. (c) PSCs after the excitation of single optical pulses in high-throughput IGZO thin films. (d) X-ray photoelectron spectroscopy O 1s profile with fitted curves for the 10-mTorr IGZO thin film (upper panel) and the ratio evolution of oxygen vacancy (V_O_) to lattice oxygen (V_L_) (lower panel). (e, f) Histogram statistics of the surface potential distribution before/after optical excitation from (e) 40-mTorr and (f) 10-mTorr IGZO thin films. (g) PSCs after the excitation of single optical pulses with different intensities for the screened 10-mTorr IGZO thin film. (h) Excitatory PSCs by optical pulse train excitations at various time intervals (Δ*t*) for the screened 10-mTorr IGZO thin films. (i) Image-recognition results for ANNs with and without image preprocessing by the screened 10-mTorr IGZO thin films. The inset shows an enlargement of the stable accuracy region.

In the first step of developing the optoelectronic co-modulated FeFETs synapses, as shown in Fig. 2a, we designed high-throughput IGZO thin films with nine gradients of growth oxygen pressures in a 3 × 3 arrangement on a SrTiO_3_ substrate. As revealed in Fig. S5, nine regions with gradient colors can be observed in the optical photograph of the deposited high-throughput IGZO thin films, in good agreement with the architectural design. The topographies of the IGZO thin films are examined by using atomic force microscopy over a randomly selected 3.0 × 3.0 µm^2^ area from each of the nine regions (Fig. 2b). All of the nine regions show smooth surfaces, with root mean square roughness values of <120 pm, indicating the excellent growth of high-throughput IGZO thin films (Fig. S6). Based on the fabricated high-throughput IGZO thin films, a high-throughput postsynaptic current (PSC) database can be built after the performance of single optical pulse excitation. As shown in Fig. 2c, action potentials are generated in all nine regions and the PSCs in the 5- and 10-mTorr regions exhibit apparent LTP synaptic characteristics, while the other regions tend to emulate short-term plasticity (STP). These results confirm that the optically modulated synaptic behavior in the IGZO thin films is highly dependent on the growth oxygen pressure. To understand the origin of the different optically modulated synaptic behaviors, a wide-scan X-ray photoelectron spectroscopy (XPS) spectrum (Fig. S7a) and V_O_ concentrations in the high-throughput IGZO thin films are investigated (Fig. S7b). By taking the regions in the 10-mTorr IGZO sample as an example, two peaks at 532.9 and 530.8 eV corresponding to an oxygen vacancy (V_O_) and lattice oxygen (V_L_) can be observed in Fig. 2d [28]. Moreover, the lower panel of Fig. 2d shows that the V_O_/V_L_ ratio increases as the growth oxygen pressure varies from 45 to 5 mTorr, suggesting that more oxygen vacancies exist in the IGZO thin films with lower growth oxygen pressure. Hence, we can infer that the higher number of oxygen vacancies in the IGZO thin films are beneficial for the realization of optically modulated LTP, which may also be consistent with the results in previous literature [33,34]. To verify this inference, scanning Kelvin probe microscopy experiments before/after optical excitation on the selected 40- and 10-mTorr regions are carried out. As shown in Fig. 2e and f, a shift of approximately +10 and +100 mV towards a high surface potential after optical excitation is observed in the 40- and 10-mTorr regions, respectively, confirming that IGZO thin-film growth under lower oxygen pressure is more sensitive to external optical excitation and suitable for the optical modulation of synaptic plasticity.

Based on the above results, we screen 10- and 5-mTorr IGZO thin films as the semiconductor layers for the optical modulation of synaptic plasticity. In the following, we choose only the 10-mTorr IGZO thin film for further study because this growth oxygen pressure is more compatible with the bilayer growth for fabricating the optoelectronic co-modulated device, as described later. Figure 2g and h shows the light-intensity-dependent PSCs and the pulse-train-stimulated PSCs, respectively, for the screened 10-mTorr IGZO thin film, suggesting that this film successfully achieves synaptic functionalities, including excitatory PSC (EPSC), STP, LTP, the STP/LTP transition, and paired-pulse facilitation (Fig. S8). With such optically modulated synaptic plasticity, the screened 10-mTorr IGZO thin film can be used to preprocess the optical images. As shown in Fig. 2i, a three-layer ANN using the screened 10-mTorr IGZO thin film for image preprocessing (details can be found in Fig. S9) achieves higher training accuracy than that without image preprocessing, suggesting the positive role of the optically modulated synaptic plasticity of this IGZO film.

**Figure 3.**
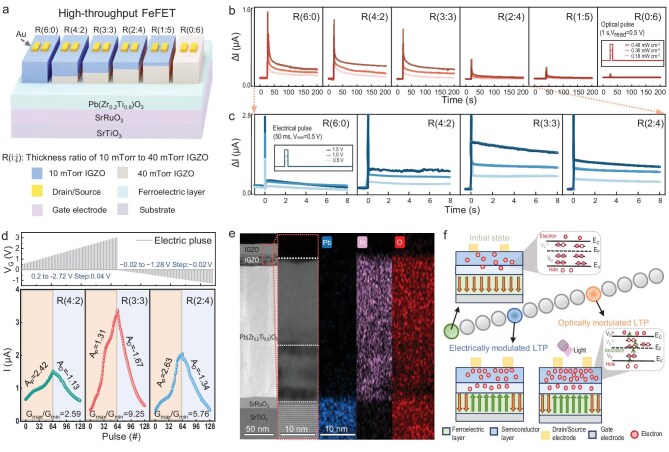
High-throughput design of the optoelectronic co-modulated FeFETs synapse. (a) High-throughput FeFETs with high-throughput thickness ratios in IGZO homostructures. (b, c) PSCs over high-throughput FeFETs after the excitation of (b) optical and (c) electrical pulses. (d) Comparison of the long-term potentiation/depression of the FeFETs devices with *R* = 6:0, 4:2, 3:3 and 2:4. (e) Cross-sectional TEM images and corresponding energy dispersive spectroscopy elemental mapping of the FeFET with *R* = 3:3. (f) Schematic illustration of optoelectronic co-modulated LTP synaptic plasticity.

However, the FeFET with the 10-mTorr IGZO thin film fails to exhibit electrically modulated synaptic plasticity, as discussed in Fig. S10. By contrast, this property is successfully achieved by the device with the 40-mTorr IGZO thin film, as shown in Fig. S11, in which the proper number of oxygen vacancies in the IGZO semiconductors can maximize the mobility and enhance the performance of the FeFETs [35,36]. Therefore, to achieve both the optical and electrical modulation of synaptic plasticity, IGZO homostructures are designed. Moreover, to optimize the performance of the IGZO-bilayered FeFET synapses, high-throughput FeFETs with six different thickness ratios in IGZO homostructures are designed. As shown in Fig. 3a, the 10- and 40-mTorr IGZO thin films are used as the upper and lower layers, respectively, to provide the FeFET synapses with optically and electrically modulable synaptic functionalities. The thickness ratio of the upper and lower IGZO layers is defined as *R*. The optically modulated synaptic functionalities of these IGZO-bilayered FeFETs are shown in Fig. 3b. PSCs corresponding to LTP synaptic plasticity can be observed in the thickness ratio range from *R* = 6:0 to *R* = 2:4, while the other thickness ratios result in the absence of the LTP synaptic plasticity. We then focus only on the four FeFETs with thickness ratios from *R* = 6:0 to *R* = 2:4 and investigate their electrically modulated synaptic functionalities. As shown in Fig. 3c, the FeFETs with thickness ratios ranging from *R* = 4:2 to *R* = 2:4 exhibit EPSC characteristics after electrical pulses. Moreover, all these three FeFETs exhibit the long-term potentiation/depression behavior (Fig. 3d). Among them, the FeFET with the thickness ratio of *R* = 3:3 achieves the largest switching ratio and the largest linear conductance change. Hence, as can be seen from Fig. 3b–d, the FeFET with *R* = 3:3 exhibits the best optoelectronic co-modulated synaptic characteristics. To explore the mechanism of the optoelectronic co-modulation in this FeFET, cross-sectional transmission electron microscopy (TEM) images of the FeFETs with *R* = 3:3 are taken. As shown in Fig. 3e, a IGZO homostructure is clearly observed in the TEM image and the energy dispersive spectrometry (EDS) mapping further suggests that the upper and lower IGZO layers have different numbers of oxygen vacancies. Based on these results, the mechanism of LTP synaptic plasticity under both electrical and optical excitations can be understood, as schematically shown in Fig. 3f. In the initial state, the downward ferroelectric polarization reduces the electron concentration in the IGZO homostructure channel, while most oxygen vacancies in the IGZO are in deep localized states (V_O_) and the remainder are in shallow donor states (V_O_^+^ and V_O_^2+^), leading to insufficient ionization of the oxygen vacancies to generate electrons [37]. These two factors result in the low conductivity of the FeFET synapse. Under the excitation of electrical pulses, partial ferroelectric polarization switches towards the IGZO homostructure, resulting in an accumulation of electrons and an increase in the conductivity of the semiconductor channel [38]. Under the excitation of optical pulses, electrons absorb photon energy to excite the valence band (E_V_) to the conduction band (E_C_) [39], while abundant electrons are also generated by the electron ionization of oxygen vacancies (V_O_ → V^1+^ + e^−^ or V_O_ → V_O_^2+^ + 2e^−^) [37]; these two mechanisms may contribute to the persistence of photoconductivity and result in the optical modulation of LTP synaptic functionalities.

**Figure 4.**
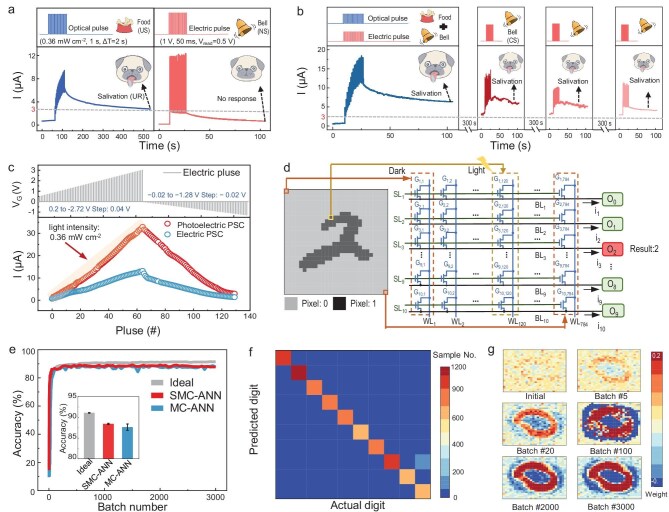
Intelligent the SMC system based on optoelectronic FeFETs. (a) Left: EPSC after electrical pulse stimulation, which produced no salivation response in a dog. Right: EPSC after a train of optical pulse excitation, which produces a salivation response in a dog. (b) Training by optoelectronic pulses and salivation response after an electrical pulse (every 300 s later). (c) Long-term potentiation/long-term depression behavior of FeFETs under electrical and optoelectronic excitation. (d) Designed architecture of the SMC–ANN. (e) Recognition accuracy of the SMC–ANN, MC–ANN and ideal ANN (based on floating-point arithmetic). (f) Confusion matrix obtained after SMC–ANN training. (g) Weight heat corresponding to output neuron ‘0’ during SMC–ANN training.

To examine the application of the optoelectronic co-modulated FeFETs, the simulation of the classical conditioned reflex is carried out by using the Pavlov’s dog experiment [40]. As shown in Fig. 4a and b, electrical pulses are used to simulate a bell (conditioned stimulus) for a dog to trigger a conditioned response, while light pulses are defined as food (unconditioned stimulus) to cause salivation (unconditioned response). As shown on the right of Fig. 4a, after 16 electrical pulses are applied (i.e. bell ringing), the PSC remains below the threshold line (dotted line), indicating that the dog does not salivate during the conditioned bell stimulation. However, the application of a train of light pulses leads to a PSC above the threshold line, indicating that salivation can be achieved by using the unconditioned food stimulation (left of Fig. 4a). The training process for the dog is shown on the left of Fig. 4b, where both optical and electrical pulses are used to simulate the association training of food and bell ringing, establishing an associative reflex between food and bell ringing with a high PSC. After training, as shown in the right parts of Fig. 4b, the PSC is above the threshold value after 16 electrical pulses are applied. Moreover, the PSC gradually decreases but remains above the threshold value when the electrical pulse train is applied repeatedly every 300 s, which is consistent with the typical findings of the Pavlov’s dog experiment [41]. These electrical and optical associative reflex dynamic responses in the Pavlov’s dog experiment confirm the application prospects for the optoelectronic co-modulated synapses.

Furthermore, light-field-assisted optoelectronic long-term potentiation/long-term depression behavior is recorded, as shown in Fig. 4c. Long-term potentiation/long-term depression under optoelectronic excitation shows better linearity and a larger ON/OFF ratio than that under electrical excitation. To achieve real-time image perception and recognition, we construct an integrated sensing–memory–computing artificial neural network (SMC–ANN) based on optoelectronic co-modulated FeFET synapses. The input images are from the Modified National Institute of Standards and Technology dataset. Each image consists of 28 × 28 pixels, with the pixel values binarized to 0 or 1. The images are labeled with the numbers 0 to 9. The architecture of the SMC–ANN has a total of 784 pixels (in agreement with the input image) and 10 devices per pixel (Fig. 4d). Each device is numbered G_i, j_, where i and j denote the sub-pixel and pixel numbers, respectively. The devices with the same number i among different pixels are connected in parallel and their output currents are summarized into the same BL_i_. The SMC–ANN adopts an in-sensor computing method. When an image is inputted, the device in the ‘1’ pixel is illuminated, while the device in the ‘0’ pixel is not illuminated. The output currents of BL_1_ to BL_10_ are compared and the one exhibiting the highest current corresponds to the prediction result. The SMC–ANN is trained online, which is described in detail in the Methods section in Fig. S12. For comparison, we design an integrated memory–computing artificial neural network (MC–ANN) based on the same FeFET synapses, but the FeFET synapses operate only with the electrical modulation in the dark. Specifically, when the MC–ANN is trained online, the weights of the FeFETs synapses are modified through long-term potentiation/long-term depression curves under only electrical excitation. Also noteworthy is that this MC–ANN uses additional photoelectric sensors to convert the input image into voltage signals, which are then fed into the device array for calculation. Figure 4e shows a comparison of the results of the SMC–ANN, MC–ANN and an ideal floating-point-based ANN. The recognition accuracy of the SMC–ANN is 88.42%, which is slightly higher than that of the MC–ANN (87.63%). The inset in Fig. 4e shows that the SMC–ANN fluctuations are smaller and converge better than those of the MC–ANN. Notably, the linearity of the conductance tuning of the long-term potentiation/long-term depression curve under electrical excitation in our FeFETs is good and, hence, the recognition accuracy of the MC–ANN is not significantly lower than that of the SMC–ANN. However, because the SMC–ANN can simultaneously sense and recognize images, whereas the MC–ANN must use additional photoelectric sensors, the SMC–ANN thus has many advantages over the MC–ANN, such as less hardware overheads, faster speed and lower power consumption. The confusion matrix obtained by using the trained SMC–ANN is shown in Fig. 4f, in which most numbers are correctly classified, resulting in the high recognition accuracy of the SMC–ANN. The weight heat map corresponding to the output neuron ‘0’ during the SMC–ANN training is shown in Fig. 4g. As the training process progresses, the weight distribution gradually converges and the eventual weight distribution successfully reflects the features of the number ‘0’, confirming that the training process of the SMC–ANN has been successfully implemented. These results confirm the robust high performance of the SMC system based on the optoelectronic co-modulated FeFET synapses in image perception and recognition.

## CONCLUSIONS

In summary, a high-throughput strategy for designing optoelectronic co-modulated ferroelectric synapses was proposed. Based on the high-throughput deposited IGZO thin films and constructed high-throughput IGZO thin-film database, we found that the IGZO thin film deposited under 10 mTorr showed excellent synaptic plasticity under optical excitation. Furthermore, a high-performance optoelectronic co-modulated FeFET synapse with an IGZO semiconductor homostructure was screened from a set of high-throughput FeFETs devices, in which the IGZO homostructure was constructed by using an upper 10-mTorr layer and a lower 40-mTorr layer at a thickness ratio of 3:3. By utilizing optoelectronic co-modulated FeFET synapses, an SMC system with high recognition accuracy, low hardware overheads, fast speed and low power consumption was demonstrated by simulation. Our work introduced a novel strategy for designing multifunctional artificial synapses, which may represent a new paradigm in the development of high-performance SMC systems.

## METHODS

### Fabrication of the high-throughput IGZO thin films and FeFET devices

High-throughput IGZO thin films were deposited under gradient oxygen pressure ranging from 45 to 5 mTorr by using a HT-PLD system with rapid movable dual masks (RP-HT-102, purchased from Shenzhen Arrayed Materials Co., Ltd in China). The high-throughput IGZO thin films was grown on SrTiO_3_(001) substrate at 180°C. In the high-throughput FeFET devices, as shown in Fig. S4, Pb(Zr_0.2_Ti_0.8_)O_3_ thin films with ∼181.3 nm thickness were deposited on SrRuO_3_-buffered SrTiO_3_(001) substrate by using HT-PLD at 600°C and 100 mTorr, while high-throughput IGZO homostructures were grown in 1 × 6 arrangements at 180°C and Au source/drain electrodes were sputtered through a shadow mask.

### Structure and electrical property characterizations

The chemical composition of the high-throughput IGZO thin films was determined by using XPS (Escalab Xi+). Electrical and optoelectronic measurements were performed by using a semiconductor analyser (Keithley 4200A-SCS) equipped with a controlled wavelength laser. Topography and kelvin probe force microscopy mapping were performed by using an atomic force microscope system (Asylum Research Cypher-ES). The high-resolution cross-sectional TEM images and EDS pattern were acquired on a TEM system (Thermo Scientific, Themis Z).

## Supplementary Material

nwaf530_Supplemental_File

## References

[1] Gao C, Liu D, Xu C et al. Toward grouped-reservoir computing: organic neuromorphic vertical transistor with distributed reservoir states for efficient recognition and prediction. Nat Commun 2024; 15: 740. 10.1038/s41467-024-44942-8 38272878 PMC10810880

[2] Kang Y, Chen Y, Tan Y et al. Bioinspired activation of silent synapses in layered materials for extensible neuromorphic computing. J Materiomics 2023; 9: 787–97. 10.1016/j.jmat.2023.02.007

[3] Zhong Y, Tang J, Li X et al. A memristor-based analogue reservoir computing system for real-time and power-efficient signal processing. Nat Electron 2022; 5: 672–81. 10.1038/s41928-022-00838-3

[4] Liu C, Wang Y, Zhang T et al. An attention mechanism-based adaptive feedback computing component by neuromorphic ion gated MoS_2_ transistors. Adv Elect Mater 2023; 9: 2201060. 10.1002/aelm.202201060

[5] Cui B, Fan Z, Li W et al. Ferroelectric photosensor network: an advanced hardware solution to real-time machine vision. Nat Commun 2022; 13: 1707. 10.1038/s41467-022-29364-8 35361828 PMC8971381

[6] Tan H, Zhou Y, Tao Q et al. Bioinspired multisensory neural network with crossmodal integration and recognition. Nat Commun 2021; 12: 1120. 10.1038/s41467-021-21404-z 33602925 PMC7893014

[7] Choi S, Yang J, Wang G. Emerging memristive artificial synapses and neurons for energy-efficient neuromorphic computing. Adv Mater 2020; 32: 2004659. 10.1002/adma.202004659 33006204

[8] Bian J, Cao Z, Zhou P. Neuromorphic computing: devices, hardware, and system application facilitated by two-dimensional materials. Appl Phys Rev 2021; 8: 041313. 10.1063/5.0067352

[9] Wu G, Zhang X, Feng G et al. Ferroelectric-defined reconfigurable homojunctions for in-memory sensing and computing. Nat Mater 2023; 22: 1499–506. 10.1038/s41563-023-01676-0 37770677

[10] Zhong G, Zi M, Ren C et al. Flexible electronic synapse enabled by ferroelectric field effect transistor for robust neuromorphic computing. Appl Phys Lett 2020; 117: 092903. 10.1063/5.0013638

[11] He J, Chen Q, Wu Y et al. Revealing fast negative capacitance in PbZr_0. 2_Ti_0. 8_O_3_ thin film with acicular ferroelastic domains. Nano Lett 2024; 24: 12426–32. 10.1021/acs.nanolett.4c02961 39351874

[12] Ren C, Zhong G, Xiao Q et al. Highly robust flexible ferroelectric field effect transistors operable at high temperature with low-power consumption. Adv Funct Mater 2020; 30: 1906131. 10.1002/adfm.201906131

[13] Ren C, Dai L, Tan C et al. Observing suppressed polarization in flexible ferroelectric negative capacitance field effect transistors. J Materiomics 2024; 10: 762–9. 10.1016/j.jmat.2023.09.008

[14] Mulaosmanovic H, Breyer E T, Dünkel S et al. Ferroelectric field-effect transistors based on HfO_2_: a review. Nanotechnology 2021; 32: 502002. 10.1088/1361-6528/ac189f 34320479

[15] Song C M, Kim D, Lee S et al. Ferroelectric 2D SnS_2_ analog synaptic FET. Adv Sci 2024; 11: 2308588. 10.1002/advs.202308588 PMC11040367 38375965

[16] Gong J, Wei Y, Wang Y et al. Brain-inspired multimodal synaptic memory via mechano–photonic plasticized asymmetric ferroelectric heterostructure. Adv Funct Mater 2024; 34: 2408435. 10.1002/adfm.202408435

[17] Li Q, Wang S, Li Z et al. High-performance ferroelectric field-effect transistors with ultra-thin indium tin oxide channels for flexible and transparent electronics. Nat Commun 2024; 15: 2686. 10.1038/s41467-024-46878-5 38538586 PMC10973520

[18] Si M, Saha A K, Gao S et al. A ferroelectric semiconductor field-effect transistor. Nat Electron 2019; 2: 580–6. 10.1038/s41928-019-0338-7

[19] Wang Z, Zhou X, Liu X et al. Van der Waals ferroelectric transistors: the all-round artificial synapses for high-precision neuromorphic computing. Chip 2023; 2: 100044. 10.1016/j.chip.2023.100044

[20] Liu Y, Wang T, Xu K et al. Low-power and high-speed HfLaO-based FE-TFTs for artificial synapse and reconfigurable logic applications. Mater Horiz 2024; 11: 490–8. 10.1039/D3MH01461D 37966103

[21] Kim I J, Kim M K, Lee J S. Highly-scaled and fully-integrated 3-dimensional ferroelectric transistor array for hardware implementation of neural networks. Nat Commun 2023; 14: 504. 10.1038/s41467-023-36270-0 36720868 PMC9889761

[22] Wan H, Zhao J, Lo L W et al. Multimodal artificial neurological sensory–memory system based on flexible carbon nanotube synaptic transistor. ACS nano 2021; 15: 14587–97. 10.1021/acsnano.1c04298 34472329

[23] Chen Z, Li W, Fan Z et al. All-ferroelectric implementation of reservoir computing. Nat Commun 2023; 14: 3585. 10.1038/s41467-023-39371-y 37328514 PMC10275999

[24] Xu M, Chen X, Guo Y et al. Reconfigurable neuromorphic computing: materials, devices, and integration. Adv Mater 2023; 35: 2301063. 10.1002/adma.202301063 37285592

[25] Hao Q, Li P, Liu J et al. Bandgap engineering of high mobility two-dimensional semiconductors toward optoelectronic devices. J Materiomics 2023; 9: 527–40. 10.1016/j.jmat.2022.11.009

[26] Hong S, Cho H, Kang B H et al. Neuromorphic active pixel image sensor array for visual memory. ACS nano 2021; 15: 15362–70. 10.1021/acsnano.1c06758 34463475

[27] Gao J, Lian X, Chen Z et al. Multifunctional MoTe_2_ Fe-FET enabled by ferroelectric polarization-assisted charge trapping. Adv Funct Mater 2022; 32: 2110415. 10.1002/adfm.202110415

[28] Tang M, Dai L, Cheng M et al. High-throughput screening thickness-dependent resistive switching in SrTiO_3_ thin films for robust electronic synapse. Adv Funct Mater 2023; 33: 2213874. 10.1002/adfm.202213874

[29] Su P, Ren C, Zeng L et al. High-throughput exploration of phase evolution in (Pb_1−X_Ba_X_) ZrO_3_ thin films. Adv Elect Mater 2024; 10: 2300746. 10.1002/aelm.202300746

[30] Yan J, Dai L, Tang M et al. High-throughput screening ferroelectric-dielectric heterostructure for robust memristor and artificial synapse. IEEE Trans Electron Devices 2025; 72: 5736–40. 10.1109/TED.2025.3599826

[31] Yin Z, Shan L, Ci R et al. Visible light-driven synaptic transistors based on bilayer InGaZnO homojunction for neuromorphic computing. Appl Phys Lett 2025; 126: 103302. 10.1063/5.0256082

[32] Song J, Meng J, Lu C et al. Photoelectric synaptic device based on bilayer OR/OP-InGaZnO for neuromorphic computing. IEEE Electron Device Lett 2024; 45: 120–3. 10.1109/LED.2023.3335628

[33] Jeon S, Ahn S E, Song I et al. Gated three-terminal device architecture to eliminate persistent photoconductivity in oxide semiconductor photosensor arrays. Nat Mater 2012; 11: 301–5. 10.1038/nmat3256 22367002

[34] Ahn S E, Song I, Jeon S et al. Metal oxide thin film phototransistor for remote touch interactive displays. Adv Mater 2012; 24: 2631–6. 10.1002/adma.201200293 22499356

[35] Lee J Y, Tarsoly G, Choi S G et al. Influences of oxygen plasma posttreatment on electrical characteristics of amorphous indium–gallium–zinc–oxide thin-film transistors. Physica Status Solidi (a) 2021; 218: 2100205. 10.1002/pssa.202100205

[36] Kim K, Park S Y, Lim K H et al. Low temperature and solution-processed Na-doped zinc oxide transparent thin film transistors with reliable electrical performance using methanol develop and surface engineering. J Mater Chem 2012; 22: 23120–8. 10.1039/c2jm33790h

[37] Kim M K, Lee J S. Synergistic improvement of long-term plasticity in photonic synapses using ferroelectric polarization in hafnia-based oxide–semiconductor transistors. Adv Mater 2020; 32: 1907826. 10.1002/adma.201907826 32053265

[38] Toprasertpong K, Takenaka M, Takagi S. Direct observation of interface charge behaviors in FeFET by quasi-static split CV and Hall techniques: revealing FeFET operation. IEEE IEDM Tech Dig 2019; pp. 23.7.1–4.

[39] Lee M, Lee W, Choi S et al. Brain-inspired photonic neuromorphic devices using photodynamic amorphous oxide semiconductors and their persistent photoconductivity. Adv Mater 2017; 29: 1700951. 10.1002/adma.201700951 28514064

[40] Bannur B, Kulkarni G U. On synapse intelligence emulated in a self-formed artificial synaptic network. Mater Horiz 2020; 7: 2970–7. 10.1039/D0MH01037E

[41] Han S, Ma T, Li H et al. Photoferroelectric perovskite synapses for nneuromorphic computing. Adv Funct Mater 2024; 34: 2309910. 10.1002/adfm.202309910

