# Supplementary material for: High-throughput design of optoelectronic–ferroelectric heterostructure from materials to sensor–memory–computing devices
Source: Natl Sci Rev. 2025 Nov 26;13(2):nwaf530. doi: 10.1093/nsr/nwaf530 (PMC12839522; doi:10.1093/nsr/nwaf530)
Supplement: nwaf530_Supplemental_File [file nwaf530_supplemental_file.pdf]

Supplementary Information

High-Throughput Design of Optoelectronic-Ferroelectric Heterostructure from Materials to Sensor–Memory–Computing Devices

Gaokuo Zhong[a,b,1,*], Jiaqi Yan[b,c,1], Mingkai Tang[b,c,1], Haoyue Deng[d], Yangchun Tan[b,c], Xiangli Zhong[c], Changjian Li[e], Zhen Fan[d,*], Jinbin Wang[c,*], Jiangyu Li[e*]

a. Changsha Semiconductor Technology and Application Innovation Research Institute, College of Semiconductors (College of Integrated Circuits), Hunan University, Changsha, China

b. Shenzhen Institute of Advanced Technology, Chinese Academy of Sciences, Shenzhen, Guangdong, China.

c. National-Provincial Laboratory of Special Function Thin Film Materials, School of Materials Science and Engineering, Xiangtan University, Xiangtan, Hunan, China.

d. Institute for Advanced Materials, South China Academy of Advanced Optoelectronics, South China Normal University, Guangzhou, China.

e. Department of Materials Science and Engineering, Southern University of Science and Technology, Shenzhen, Guangdong, China.

[1] These authors contributed equally to this work.

[*] Authors to whom the correspondence should be addressed to: gkzhong@hnu.edu.cn, fanzhen@m.scnu.edu.cn, jbwang@xtu.edu.cn, and lijy@sustech.edu.cn.

Keywords: Ferroelectric; Ferroelectric field-effect transistors; High-throughput; Artificial synapses; Sensing-memory-computing integration.

Supplementary Figures

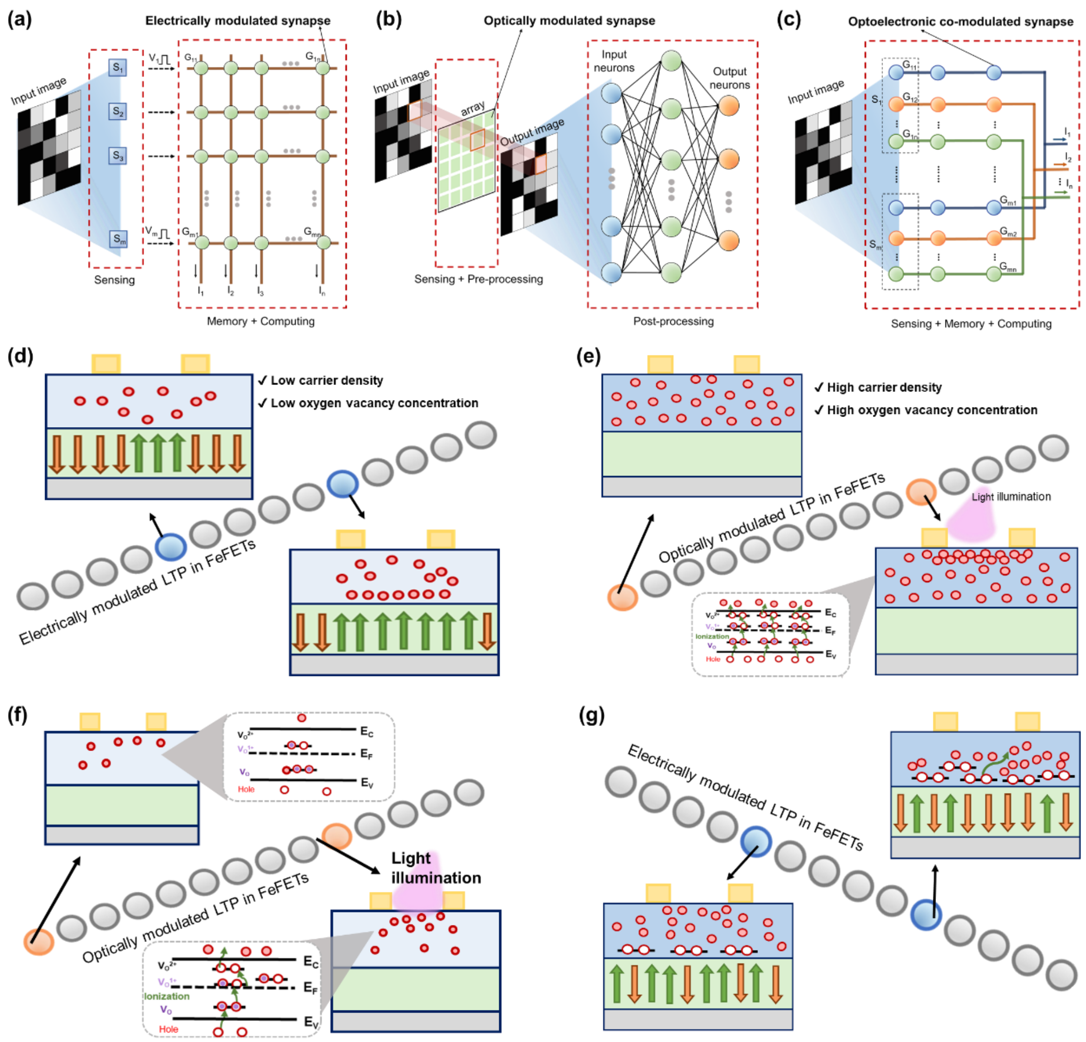

Supplementary Figure 1: Optoelectronic co-modulated synapse. Schematic illustrations of (a) a MC system based on electrically modulated synapses, (b) a neuromorphic vision sensor based on optically modulated synapses, and (c) an SMC system based on optoelectronic co-modulated synapses. (d) Mechanisms of electrically modulated long-term potentiation (LTP) synaptic plasticity in FeFET synapses, and (e) optically modulated LTP synaptic plasticity in FeFET synapses. (f) The potentiel explain of the FeFET can not optically modulated LTP synaptic plasticity. (g) Possible reason for the FeFETs based on a semiconductor layer with a high oxygen vacancy concentration and a high carrier density failing to exhibit electrically modulated LTD synaptic plasticity.

Parallel data processing and high-accuracy recognition have been achieved via the construction of artificial neural networks (ANNs) with an integrated memory–computing (MC) architecture (Fig. S1a). However, to process visual information, such MC systems require additional photoelectric sensors to convert light signals into electrical signals. Alternatively, neuromorphic vision sensors with optically modulated synapses enable the preprocessing of visual information (Fig. S1b), but postprocessing units are needed to further process the data. From an in-sensor computing perspective, if FeFETs based synapses are both electrically and optically modulatable, they can be used to construct ANNs with an integrated sensor–memory–computing (SMC) architecture. Such SMC systems can realize the real-time perception and processing of visual information directly in the sensor (Fig. S1c), which can greatly reduce the hardware overhead, time latency, and energy consumption.

The potentiel explain of the FeFET can not optically modulated LTP synaptic plasticity. Why the FeFET based on a semiconductor layer with a low oxygen vacancy concentration and a low carrier density fails to exhibit optically modulated LTP synaptic plasticity is explained in Fig. S1d-e. Such a semiconductor layer can generate only a limited number of carriers under optical excitation. In addition, the lack of oxygen vacancies causes the absence of the persistent photoconductivity. Therefore, the optically modulated LTP synaptic plasticity is not achieved in this FeFETs. Why the FeFET based on a semiconductor layer with a low oxygen vacancy concentration and a low carrier density fails to exhibit optically modulated LTP synaptic plasticity is explained in Fig. S1f. Such a semiconductor layer can generate only a limited number of carriers under optical excitation. In addition, the lack of oxygen vacancies causes the absence of the persistent photoconductivity. Therefore, the optically modulated LTP synaptic plasticity is not achieved in this FeFETs. Why the FeFET based on a semiconductor layer with a high oxygen vacancy concentration and a high carrier density fails to exhibit electrical modulated LTD synaptic plasticity is explained in Fig. S1g. The downward polarization induced by negative voltage pulses fail to completely deplete the carriers in the semiconductor layer due to the high carrier density. In addition, the oxygen vacancies can screen the polarization charge, further contributing to the failure of LTD synaptic performance.

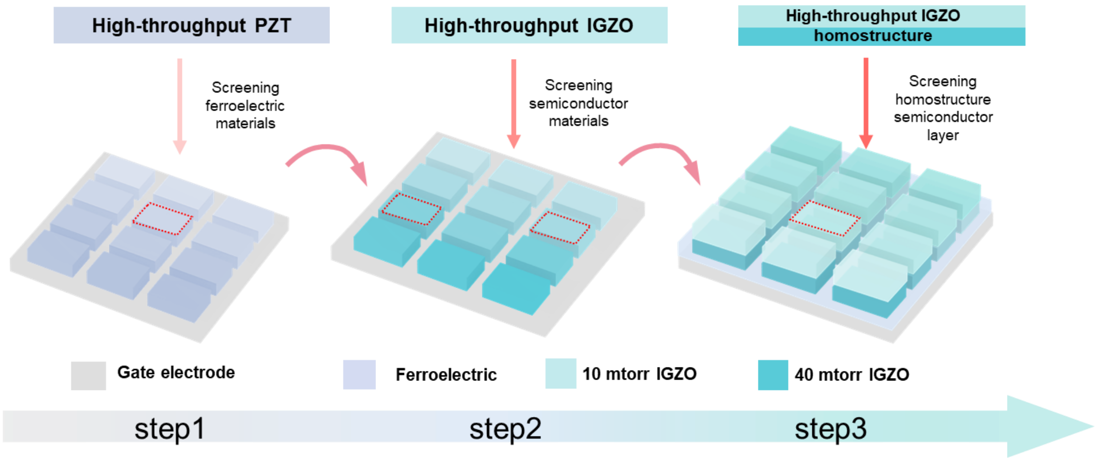

Supplementary Figure 2: Flow chart of the multi-step high-throughput strategy of optoelectronic co-modulated FeFETs.

As shown in Fig. S2, the multi-step high-throughput strategy can be divided into three major stages: (i) screening of ferroelectric PZT layer, (ii) screening of semiconductor IGZO layer, and (iii) screening of IGZO -based FeFETs. Each step are details described as follow:

(i) Step 1: High-throughput PZT thin films with varying thicknesses were fabricated to identify ferroelectric parameters that ensure robust polarization switching, which is essential for FeFET operation.

(ii) Step 2: High-throughput IGZO thin films were deposited under different oxygen pressures, and a database of post-synaptic currents (PSCs) was established. This step enabled the screening of IGZO layers with suitable optical and electrical responses for optoelectronic synaptic modulation.

(iii)Step 3: High-throughput FeFETs incorporating IGZO heterostructures with different thickness ratios were evaluated, leading to FeFET synapses capable of dual optical and electrical modulation.

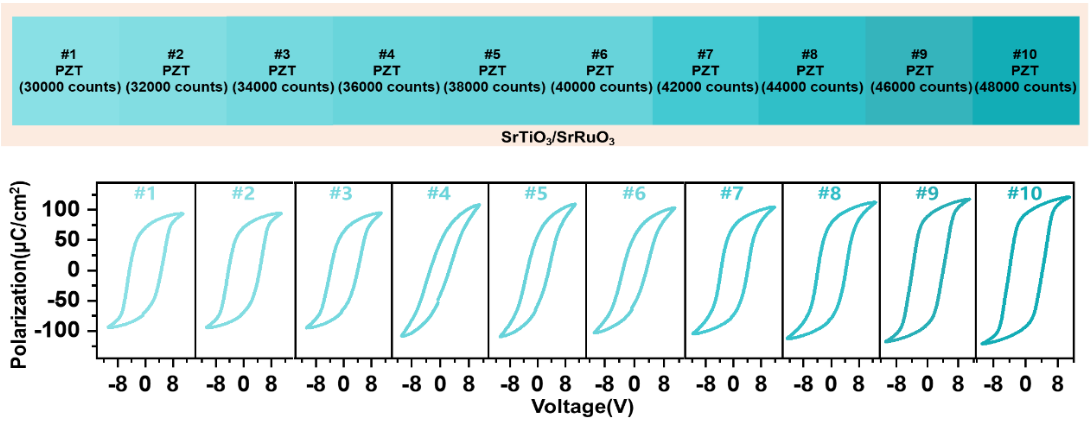

Supplementary Figure 3: The *P-E* curves of high-throughput PZT films under different thicknesses (Different laser count)

As shown in Fig. S3, high-throughput screening of PZT films with different thicknesses (controlled by varying laser pulse numbers from 30,000 to 48,000). A progressive increase in coercive voltage and saturation polarization is observed, which allows for determining the optimized PZT thickness that provides maximum polarization under the lowest possible switching voltage.

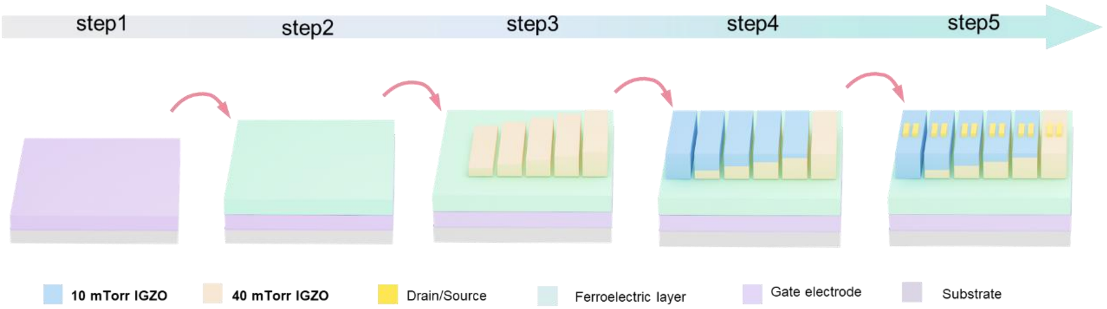

Supplementary Figure 4: Flow chart of the construction process of the high-throughput heterostructure

As shown in Fig. S4, specific steps are as follows:

(i) **Step 1:** Deposition of bottom electrodes: $SrRuO_3$ (SRO) bottom electrodes were first deposited on $SrTiO_3$ (STO) substrates.

(ii) **Step 2:** Growth of ferroelectric layer: PZT thin films were subsequently grown on the SRO/STO structure, with the deposition parameters optimized through high-throughput screening.

(iii) **Step 3:** Deposition of IGZO base layer: An IGZO base layer was deposited under an oxygen pressure of 10 mTorr, with its thickness systematically varied.

(iv) **Step 4:** Deposition of IGZO upper layer: An additional IGZO layer was deposited on top of the base layer under 40 mTorr oxygen pressure, with different thicknesses, thereby forming the high-throughput IGZO heterostructure.

(v) **Step 5:** Formation of source/drain electrodes: Au source and drain electrodes were finally deposited on the IGZO heterostructure to complete the device fabrication.

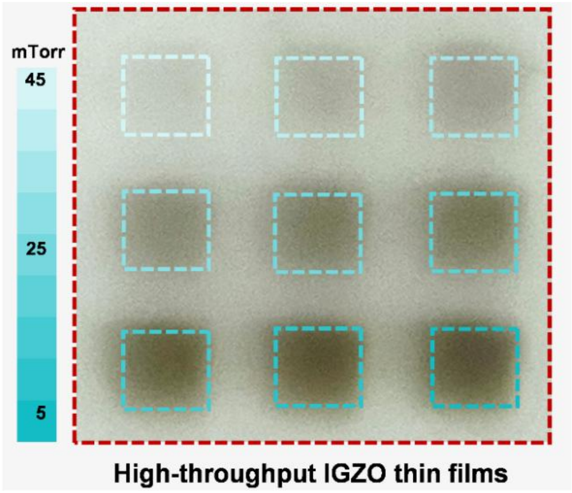

Supplementary Figure 5: the images of the fabricated high-throughput IGZO thin films with different growth oxygen pressure.

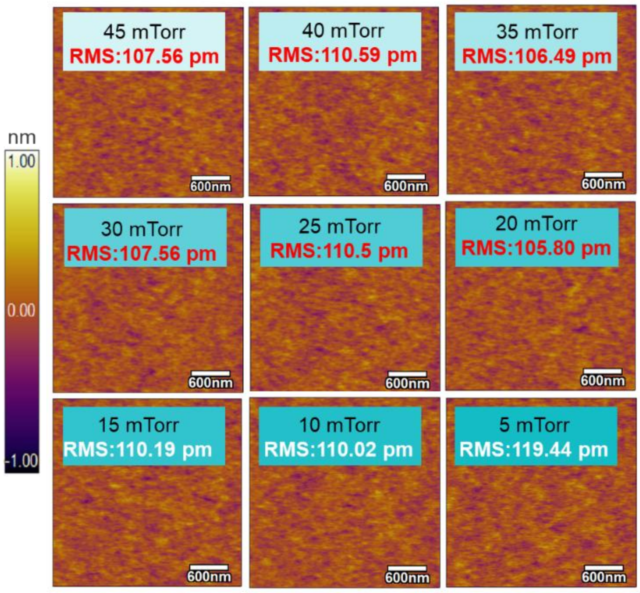

Supplementary Figure 6: topography of these high-throughput IGZO thin films with evaluated root-mean-square (RMS) roughness values.

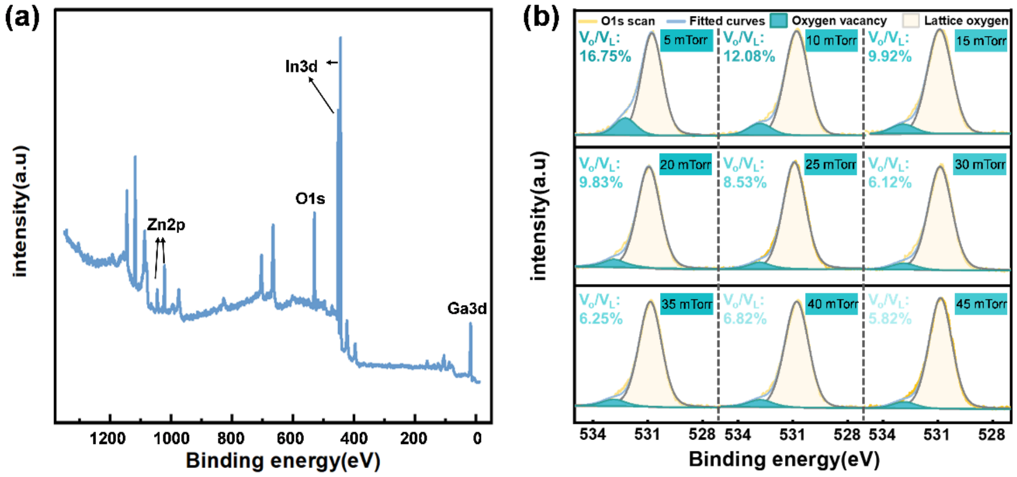

Supplementary Figure 7: The wide-scan XPS spectrum of a IGZO thin films with 10 mTorr growth oxygen pressure. XPS O1s profile with fitted curves from the high-throughput IGZO thin films.

As shown in Fig. S7a, the peaks at 18 eV, 427 eV-429 eV, 531 eV,1040 eV-1050 eV correspond to Ga3d, In3d, O1s, Zn2p, respectively. The V_O concentrations in the nine regions with different IGZO OP values (gradient increased from 5 to 45 mTorr) were estimated via X-ray photoelectron spectroscopy (XPS). As shown in Fig. S7b, two peaks at 532.9 and 530.8 eV correspond to V_O and V_L, respectively. Moreover, the V_O /V_L ratios were estimated using the fitted peak areas as 0.0582, 0.0682, 0.0625, 0.0612, 0.0853, 0.0983, 0.0992, 0.1208, and 0.1675 for the nine gradient IGZO, respectively. It can be inferred that with the decrease of growth oxygen pressure, the oxygen vacancy ratio increases.

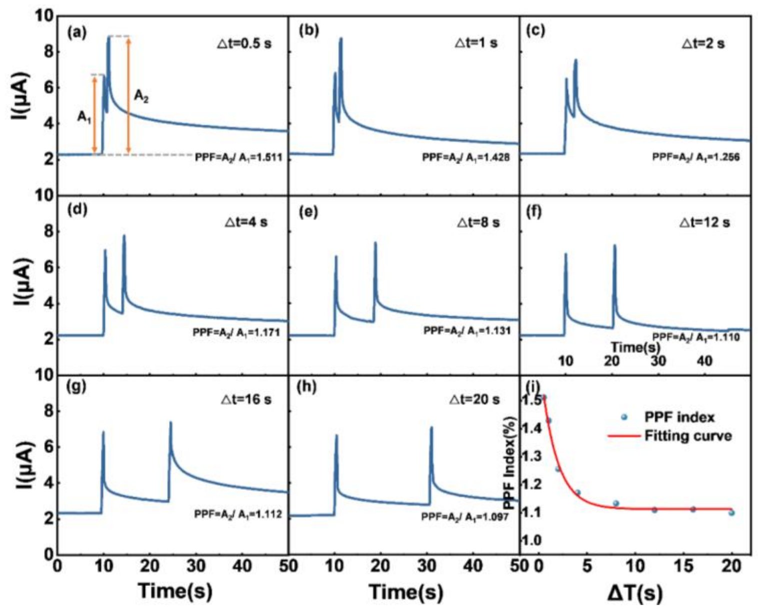

Supplementary Figure 8: (a-h) The Paired-pulse facilitation (PPF) behavior triggered by a pair of light spikes (0.36 nW μm^{-2}, 1s) with Δt =0.5 s,1 s,2 s,4 s,8 s,12 s,16 s,20 s. (i) PPF ratio as a function of the pulse interval, defined as $(A_2 - A_1)/A_1 \times 100\%$, where the red line represents fitting results using the double exponential decay function.

Fig. S8a shows an output waveform, where the base current is around 0.2 μA, and A_1 and A_2 represent the amplitudes after the first and second laser pulses. The second pulse exhibits a stronger response than the first pulse, showing typical Paired-pulse facilitation (PPF) characteristics. As shown in Fig. S8a-h, with the Δt increases, the post-synaptic current elicited by the second light pulse decreases and exhibits a reduction in the PPF. In Fig. S8i, the red line here indicates the fitting curve at the experimental points, which obeys an exponential decay, consistent with the theoretical result. All of these are consistent with the memory behavior of biological synapses.

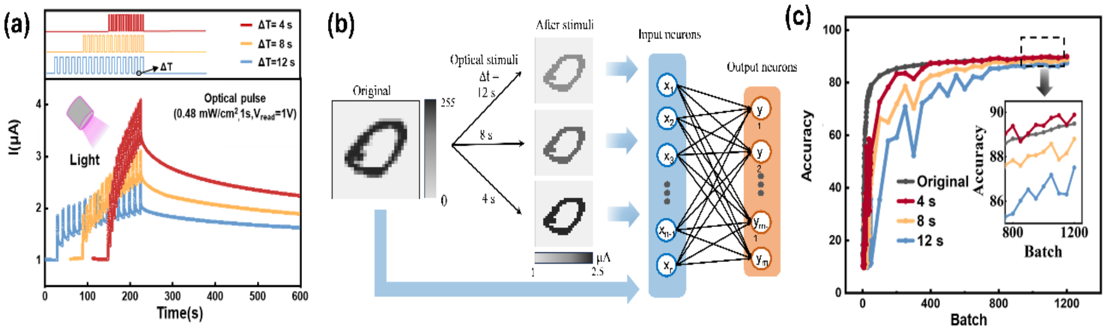

Supplementary Figure 9: (a) Excitatory PSC by a train of optical pulses excitation with varied time intervals (Δt) for the screened 10 mTorr IGZO thin film. (b) Schematics illustrating the image contrast enhancement implemented by using the screened 10 mTorr IGZO thin films.

The optically excited PSC behavior suggests that the 10 mTorr IGZO thin film can be used to implement image contrast enhancement, as illustrated in Fig. S9b. The input images are the MNIST handwritten digit images, consisting of 28 × 28 pixels. The pixel values are binarized to 0 or 1. In each pixel, there is a 10 mTorr IGZO thin film-based optical synapse. The device in the "1" pixel is illuminated by 16 optical pulses with 0.36 mW/μm^2 power and different Δt (4 s, 8 s, and 12 s), while the device in the "0" pixel is not illuminated. After this preprocessing, the currents (read at 400 s) of all the devices can constitute a preprocessed image, as shown in the middle of Fig. S9b. It is seen that the image after preprocessing by using the optical pulses with Δt = 4 s shows the highest contrast. This can be explained by the fact that the highest current is retained after applying optical pulses with Δt = 4 s (see Fig. S9a). Then, all these preprocessed images together with the original image are sent to a three-layer artificial neural network (ANN) with 784 input neurons, 128 hidden neurons, and 10 output neurons to perform the classification. Fig. S9c shows the classification results after image preprocessing by using the optical pulses with different Δt. It is seen that the accuracy corresponding to Δt = 4 s is the highest, well attributed to the highest image contrast after preprocessing by using the optical pulses with Δt = 4 s. Note that only the results corresponding to original and Δt = 4 s in Fig. S9c are reported in Fig. 2i in the main text.

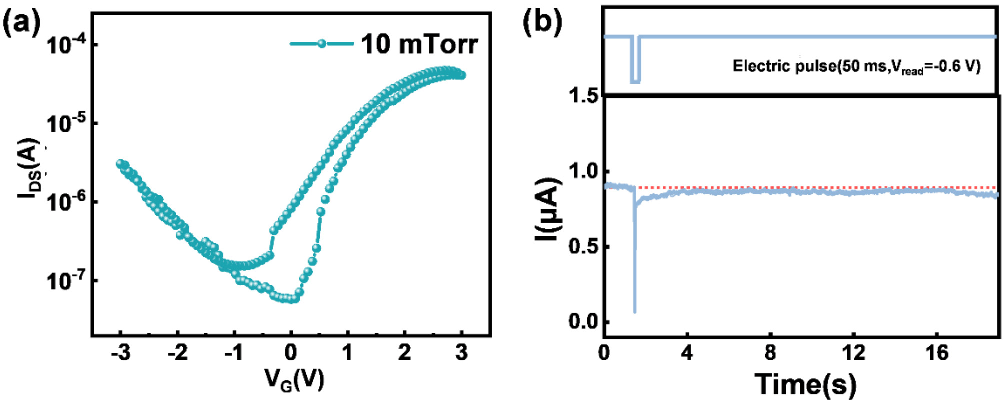

Supplementary Figure 10: (a) The transfer curve of FeFET with 10 mTorr IGZO. (b) PSC after the excitation of negative electrical pulses.

As can be observed from the transfer curve of FeFETs in Fig.10, the source-drain current of FeFET with the 10 mTorr IGZO thin film gradually increases as the negative gate voltage decreases, indicating that the device cannot be in the off-state and making this FeFETs fails to exhibit the electrical modulated synaptic plasticity (as shown in Fig. S10a). Such performance may results from the charge trapping and localized defect states arised by large amount of oxygen vacancies in 10 mTorr IGZO thin film.

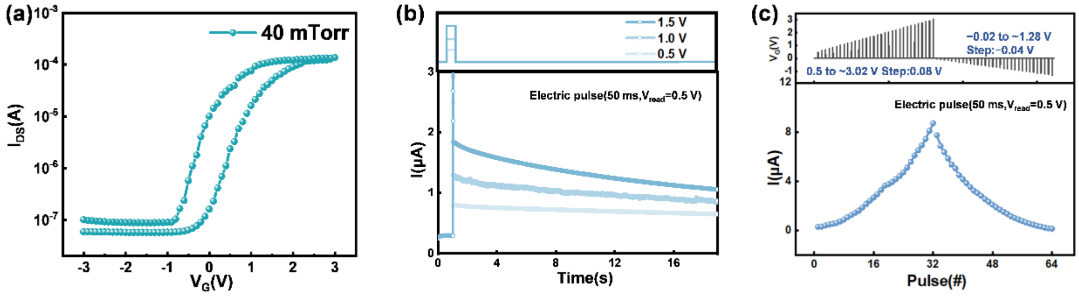

Supplementary Figure 11: (a) The transfer curve of FeFET with 40 mTorr IGZO. (b) PSC after the excitation of electrical pulses. (c) Long-term potentiation/long-term depression behavior of FeFET with 40 mTorr IGZO.

The transfer curve of FeFET with 40 mTorr IGZO is shown in Fig. S11a, where a steep switching in the transistor characteristic and a memory window of 1.2 V is observed for the bidirectional gate voltage sweep of 3 V. As shown in Fig. S11b, when the intensity of electric pulses is increased from 0.5 V to 1.5 V with a fixed pulses width of 50 ms, the FeFET synapse exhibits LTP synaptic characteristic with enhanced EPSC, demonstrating the ability of electrical regulation of conductance. The long-term

potentiation/long-term depression behavior is shown Fig. S11c, the FeFET synapse show good synaptic plasticity with large switching ratio (G_{max}/G_{min}=18.3) and nonlinearity of the conductance change (A_P=-1.87, A_D=-1.35).

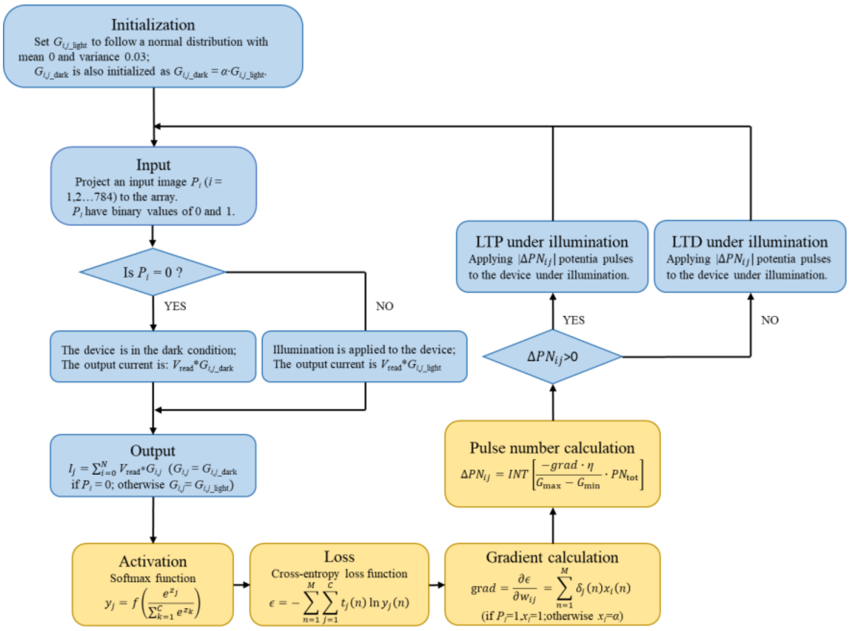

Supplementary Figure 12: Schematic diagram illustrating the online training method for the SMC-ANN based on our optoelectronic co-modulated FeFET synapses.

The online training process for SMC-ANN based on our optoelectronic co-modulated FeFET synapses is as follows. First, the forward propagation process is performed. When an image is inputted, the devices in "1" pixels are illuminated, resulting in an output photocurrent; while the devices in "0" pixels are not illuminated, resulting in a dark current output. At this stage, the summed currents from BL_1 to BL_{10} are read, and the label of the BL that has the highest current represents the predicted digit. Then, the backward propagation process is executed. The weight update amount is calculated based on the difference between the predicted result and the actual result, and then converted into spike numbers and applied to the corresponding devices. At this point, all devices are illuminated, so the weight updates follow the LTP/LTD curve under illumination. Subsequently, the next round of forward propagation begins. As mentioned earlier, if a device is illuminated, its conductance value is the updated photoconductance (i.e., the conductance obtained from the LTP/LTD curve under illumination), and the output photocurrent is calculated based on this photoconductance

value. If a device is not illuminated, its conductance value is the dark conductance associated with the updated photoconductance, and the output dark current is calculated based on this dark conductance value.
